# The Importance of Early Detection and Prevention of Atypical Skin Lesions and Other Melanoma Risk Factors in a Younger Population

**DOI:** 10.3390/cancers16244264

**Published:** 2024-12-22

**Authors:** Paulina Karp, Katarzyna Karp, Marcelina Kądziela, Radosław Zajdel, Agnieszka Żebrowska

**Affiliations:** 1 Department of Dermatology and Venereology, Medical University of Lodz, pl. Hallera 1, 90-647 Lodz, Poland; paulina.karp@umed.lodz.pl (P.K.); katarzyna.karp@stud.umed.lodz.pl (K.K.); marcelina.kadziela@stud.umed.lodz.pl (M.K.); 2 Department of Computer Science in Economics, University of Lodz, 90-237 Lodz, Poland; radoslaw.zajdel@umed.lodz.pl; 3 Department of Medical Informatics and Statistics, Medical University of Lodz, 90-645 Lodz, Poland

**Keywords:** dysplastic naevus, melanoma, risk factors, dermoscopy, prevention

## Abstract

Background/Objectives: Skin cancer is becoming increasingly common due to increasing risk factors such as excessive ultraviolet (UV) radiation, genetic predisposition, fair skin, and a history of sunburn. Melanoma accounts for only 1% of cases but causes most skin cancer deaths. Dysplastic nevi (DN) are important precursors of melanoma. The aim of this study was to investigate the influence of these risk factors on the incidence and stage of skin cancer. Methods: The study included 591 patients aged 18 to 64 who visited the Department of Dermatology and Venereology in 2022–2023 for skin examinations. Each patient completed a questionnaire regarding the risk factors for melanoma and atypical melanocytic nevi and then underwent a dermatoscopic examination of the whole body using a digital videodermatoscope. Results: Dermatoscopic examination revealed a lesion suggestive of melanoma in 1.69% of the patients. Risk factors for developing melanoma included male gender, family history of melanoma, number of skin moles, sunburn in childhood, sun-dependent hobby, using a tanning bed, using low sun protection factor (SPF) cream, not avoiding sun exposure, and co-occurrence of actinic keratosis. Conclusions: Risk factors for melanoma and dysplastic nevi are still common among patients, but the situation has been improving over the years. Early intervention and education on sun safety can play pivotal roles in reducing the incidence of atypical moles and potentially preventing malignant transformations.

## Simple Summary

Although melanoma is much less common than other skin cancers, it has a higher mortality rate and is responsible for almost 73% of skin cancer-related deaths. Dysplastic nevus (DN) is known as a key factor contributing to the development of cutaneous melanoma. Early detection and monitoring are crucial for individuals with atypical nevi. This study’s aim was to investigate the role of selected risk factors in the incidence of skin cancers and the stage of advancement at diagnosis. Our study involved a group of younger people and highlighted several key factors influencing the occurrence of atypical skin lesions. However, it also focuses attention on the significant correlation between the occurrence of atypical lesions and various clinical and demographic factors in this age group. Our findings underscore the necessity for targeted prevention strategies and regular dermatologic screening, particularly for the high-risk groups identified in this study.

## 1. Introduction

Skin cancer is one of the most common malignancies worldwide, and its incidence continues to rise. Understanding the risk factors associated with skin cancer is crucial for both prevention and early detection. These risk factors include, but are not limited to, excessive ultraviolet (UV) radiation exposure, genetic predisposition, fair skin, and a history of sunburn. Awareness and monitoring of these risk factors can significantly impact public health outcomes by enabling early interventions and reducing the burden of skin cancer. This publication highlights the importance of recognizing and tracking skin cancer risk factors to enhance preventative strategies and improve patient prognosis.

Skin cancers can be divided into two groups: melanocytic skin cancers (MSCs) and non-melanocytic skin cancers (NMSCs). Risk factors for NMSC are diverse, encompassing individual characteristics and various environmental and occupational exposures. Host susceptibility factors, such as fair skin type, light or red hair, a tendency to sunburn, family history of skin cancer, and genetic polymorphisms, play significant roles in the development of these malignancies [1].

Approximately 80% of NMSCs are basal cell carcinoma (BCC), the most common skin cancer, derived from the basal cells. Intermittent ultraviolet radiation (UVR) exposure and UVR exposure during childhood are identified as the most prominent predisposing factors. Squamous cell carcinoma (SCC) accounts for about 16% of skin cancer cases. There is a strong association between numerous factors and the incidence of SCC: cumulative habitual sun exposure, human papillomavirus, chronic scarring conditions, familial cancer syndromes, and environmental exposures, such as to arsenic [2].

Melanoma is a malignancy arising from skin melanocytes and accounts for approximately 1% of all skin cancers but is responsible for most skin cancer-related deaths. Risk factors for the development of melanoma include genetic factors, UV exposure, number of nevi, skin type, age, personal or family history of skin cancer, and immune system suppression [3]. It is a growing public health problem because the incidence of melanoma is increasing worldwide, particularly in White populations [4]. This increase is associated with the elevation of the level of sunlight exposure, a growing number of immunosuppressed people, and enhancement of life expectancy [4].

In Poland, the incidence rate of melanoma is approximately 9.6 per 100,000. According to the National Cancer Registry of Poland, 3689 (1749 men and 1940 women) cases of skin melanoma were recorded in Poland, and 1464 deaths due to it in 2019. It is estimated that in 2024 the number of cases will increase to 5129 and the number of deaths to 1964 [5]. Melanoma is much less common than other skin cancers, it has a higher mortality rate and is responsible for almost 73% of skin cancer-related deaths [6].

Dysplastic nevi (DNs) are known as an important factor contributing to the development of cutaneous melanoma. These lesions may be present in enormous amounts in patients with atypical nevus syndrome, or isolated without family occurrence. DNs usually occur in sun-exposed areas, especially the upper limbs and trunk. The ABCD (E) criteria help clinicians with making a diagnosis. A nevus is considered atypical when it is asymmetrical (A), has unequal borders (B), has multiple colors (C), diameter ≥5 mm (D), and it protrudes above the surface of the skin (E) [7].

The differentiation between atypical nevi and common nevi often relies on specific features observed under dermatoscopic examination, including the pigment network and brown globules. In atypical nevi, the pigment network tends to be irregular, with focal prominence and abrupt termination at the periphery in some areas [8]. In common nevi, the pigment network is typically regular and fades gradually toward the periphery of the lesion. In atypical nevi, brown globules may exhibit varied sizes and shapes, with an irregular distribution. In contrast, brown globules in common nevi are usually uniform in size and shape, often with regular distribution, especially in the central area of the lesion [9]. Atypical moles can resemble melanoma and are often biopsied to rule out cancer. While having multiple atypical nevi can increase the risk of developing melanoma, the likelihood of any single atypical mole transforming into melanoma is low [10,11].

Early detection and monitoring are crucial for individuals with atypical nevi due to their increased risk of melanoma. However, a dysplastic nevus is not a precursor to melanoma. This diagnosis should be used to prompt healthcare providers to recommend closer monitoring and follow-up for patients [12]. In our center, dermatoscopic diagnosis of an atypical nevus qualifies for intensified dermatoscopy of skin lesions every 3–6 months. This study aimed to investigate the role of selected risk factors in the incidence of skin cancers and precancerous lesions in the younger population.

## 2. Materials and Methods

Our study included 591 patients aged from 18 to 64 who visited the Department of Dermatology and Venereology in Lodz in 2022–2023 for a skin examination. Each patient completed a detailed questionnaire containing questions about the risk factors for the development of melanoma and atypical melanocytic nevi. Then, the patients underwent a whole-body dermatoscopic examination conducted with the aid of a digital video dermatoscope FotoFinder [13].

### Statistical Analysis

The analyses were conducted using STATISTICA version 13.3 (StatSoft Poland Inc., Tulsa, OK, USA). First, distributions of the study variables were calculated. The Shapiro–Wilk test was used to check the normality of the data. The descriptive statistical analysis included standard measures, with the arithmetic mean for numerical variables and standard deviation provided in the paper as a measure of the variability of the given features. For qualitative data, the descriptive measures were absolute counts and structure indices (percentages). Then, a statistical analysis was carried out to determine the impact of potential factors on melanoma risk. To identify predictors, univariate and multivariate logistic regression analyses (with the results presented as odds ratios (ORs) and 95% confidence intervals (95% CIs)) were performed. The variables with *p*-values of 0.05 or less from the univariate analysis were included in the multivariate model. The independent values included gender, number of lesions on skin, presence of dysplastic nevi, actinic keratosis, sunburn during sunbathing, sun-dependent hobby, using a tanning bed, sun exposure limitation, usage of skin sun protection cream; these were revealed to be, to some extent, prognostic factors in the univariate analysis, thus the mentioned variables were used in performing the multivariate model. A *p*-value below 0.05 was considered statistically significant.

## 3. Results

The study group consisted of 591 patients—393 women (66.5%) and 198 men (33.5%)—who participated in the EU project titled: Melanoma without secrets—examine the moles. Skin cancer prevention program. The mean age of the women was 46.3 (SD of 12.077) and of the men 45.2 (SD of 12.590). The most common reasons for patients to undergo dermatoscopic examination were the following: “many moles, hyperpigmentations on the skin” (64.94%), “preventive skin examination” (24.9%), “the mole has changed, or a new skin lesion has appeared” (4.91%).

Table 1 shows that 382 of the patients (64.74%) never perform skin self-examination, while 126 (21.36%) examine themselves once every six months, 43 (7.29%) once a month, 30 (5.08%) once a week, and 9 (1.53%) every day. In this population, 25 (4.23%) patients reported a family history of melanoma. Sunburn in childhood was reported by 267 patients (45.17%). Among the other risk factors for the development of melanoma, 39 (6.6%) patients reported regularly using a tanning bed, 377 patients (63.79%) patients had hobbies related to spending time in the sun, 544 patients (92.05%) declared having had at least 1 year of work involving prolonged periods of sun exposure.

Only 202 patients (34.18%) confirmed always using sunscreen during intense exposure to the sun and 40 people (6.77%) never used it, whereas always wearing sunglasses was reported in 199 patients (33.67%) and sometimes in 291 patients (49.24%). A total of 377 patients (63.79%) did not know the approximate number of moles on their body. In the study group, 471 patients (79.70%) had never had a dermatoscopic examination before. As a result of this examination, dermatologists diagnosed no neoplasm lesions in 509 patients (86.13%), lesions suggesting melanoma in 10 patients (1.69%), non-melanoma skin cancer in 20 patients (3.38%), and a suspicious lesion in 52 patients (8.8%). The demographics are summarized in Table 1.

### Factors Associated with Atypical Nevi

Table 2 reports the associations between the atypical nevi and various risk factors. Males were almost twice as likely to have atypical nevi diagnosed at the exam than females: OR= 1.996 [1.245; 3.201], *p* = 0.004. In the group of people with a larger number of moles (51–100 moles), the risk of their atypicality was 2.3 times higher than for people with a normal number of moles (OR= 2.305 [1.057; 5.027], *p* = 0.036), when the number of moles exceeded 100 or more, the probability was even higher (OR= 2.305 [1.057; 5.027], *p* = 0.036).

Participants who had an actinic keratosis were three times as likely to have an atypical mole also diagnosed (OR= 3.074 [1.213; 7.786], *p* = 0.018). Other risk factors such as sunburn during sunbathing, sun-dependent hobbies, using a tanning bed, and not avoiding sun exposure statistically increased the risk of atypical lesions in patients. In the case of using a tanning bed, the risk depended on the frequency of using it. People who never used a tanning bed had no such risk, when they used it from 1 to 20 times/year they were almost four times as likely to have an AN diagnosed: OR= 3.849 [2.129; 6.959], *p* = 0.000; when this number exceeded 20 tanning sessions/year, they were five and a half times as likely to have an AN diagnosed: OR= 5.512 [2.636; 11.525], *p*= 0.000.

People who used sun protection with a sun protection factor (SPF) >40 were not at risk of atypical moles. People who declared using SPF 21–40 had an increased risk of these moles, almost two times higher than people who used SPF >40, but the result was not statistically significant (OR = 1.999 [0.945; 4.227], *p* = 0.069). However, people who used SPF 20 or less were much more likely to have ANs.

Based on the age distribution presented in the study population, two age groups were distinguished: 18–45 years and 46 years and over. Dividing the age variable into two levels allowed us to evaluate different patient age categories for the diverse risk factors. There were 263 (44.50%) younger patients (≤45 years old) and 328 (55.50%) older ones.

**Figure 1:**
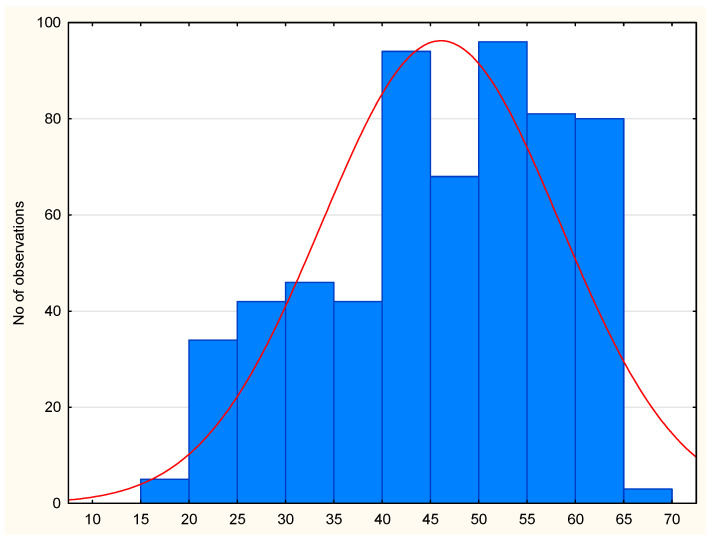
Age histogram. Shapiro–Wilk normal distribution analysis, *p* < 0.001.

Age was included as an independent variable in assessing the risk of dysplastic nevi. The impact of the variable age as a quantitative quantity was examined first. The odds ratio was 1.015 (CI95 0.995–1.035) and was statistically insignificant (*p* = 0.137) (Figure 1). The effect of age as a qualitative figure was then examined by group. Similarly, the effect was statistically insignificant (OR = 1.370, CI95 0.853–2.225, *p* = 0.190). The descriptive statistics are presented in Table 3.

## 4. Discussion

Melanocytic nevi, dysplastic nevi, and cutaneous melanoma are important interdisciplinary problems of modern medicine. Skin cancer is one of the most preventable cancers. This neoplasm develops on the surface of the body, so it can be easily noticed and diagnosed. The most common screening test is dermatoscopy, which is easy to perform, inexpensive, safe, and acceptable to patients. Moreover, if the cancer is detected at an early stage, treatment requires only local excision of the lesion [14].

Skin cancer prevention can be divided into primary, secondary, and tertiary prevention. Primary prevention aims to reduce the risk of developing skin cancer by encouraging the avoidance of known risk factors, primarily UV radiation. Minimizing exposure to UV radiation during peak sunlight can prevent sunburn. If sun exposure cannot be avoided, protective clothing, hats, sunglasses, and sunscreen are recommended [15]. It is not recommended to be exposed to the sun all day long, even when using high-SPF sunscreen. Although sunscreen prevents sunburn, it is not a surefire factor in preventing the development of melanoma [16], there is no evidence that this approach directly impacts cancer morbidity and mortality [17].

Secondary prevention focuses on increasing screening to detect precancerous and cancerous lesions early [15]. Skin cancer screening has been shown to lead to the detection of more in situ and invasive skin cancers along with more thin invasive melanomas. Furthermore, a reduction in the incidence of thick melanomas and melanoma mortality has been observed [18]. Screening of the general population is not recommended. It is necessary to identify patients at higher risk of skin cancer and they should undergo screening [17]. Tertiary prevention aims to improve the prognosis for skin cancer patients by enhancing treatment, quality of life, and recovery [14].

Over the years, in Poland and throughout Europe, many campaigns and programs have been created to increase public awareness of skin cancer, risk factors and methods of prevention. One of the largest organizations in Europe is Euromelanoma, which raises public awareness of skin cancer, provides support for screening campaigns, and educates on key preventive measures against skin cancer, risk factors, and the importance of recognizing abnormal lesions through self-examination [14]. There are also many melanoma prevention campaigns in Poland run by local organizations. One of them was the EU project titled "Melanoma without secrets—examine the moles. Skin cancer prevention program” carried out in 2022–2023. The program was dedicated to the population of younger, working people.

Prevention of melanoma and other skin cancers in younger patients is critically important, particularly before they reach retirement age. Early preventive measures can significantly reduce the risk of developing these cancers later in life. This is because cumulative exposure to ultraviolet radiation, a major risk factor for skin cancer, often begins in childhood and adolescence. By encouraging protective behaviors such as using sunscreen, wearing protective clothing, and avoiding tanning beds from an early age, the risk of skin damage and subsequent cancer can be minimized.

Educating younger populations about the dangers of UV exposure and the importance of regular skin checks is essential. Early detection of abnormal moles or skin changes can lead to prompt treatment, improving outcomes and reducing the burden of cancer in later years. Moreover, instilling these habits early creates a foundation for lifelong skin health, contributing to overall well-being and reducing healthcare costs associated with treating advanced cancers.

In this population of workers (18–64), women were more likely to undergo preventive skin examinations than men (66.5% of the responders). Our observations are confirmed by the Central Statistical Office of Poland data. According to the report from 2023, women use preventive tests more often than men [19,20]. The average age of people reporting for the study was approximately 45.5 years old.

The most frequently reported reason for undergoing a skin examination among the study population was “Many moles, hyperpigmentations on the skin”. An increased number of moles is a known risk factor for developing melanoma: there is a 1.5 times higher risk in people with 11 to 25 nevi and this doubles with every increase of 25 nevi [6]. The increased incidence of dermatoscopic examination in people with a large number of moles appears to be related to educational campaigns disseminating knowledge about risk factors. In the study by Dubbini et al., a similar question was asked and the most common answer given was preventive examination (51.5%) [21]. This Italian study was conducted from 2010 to 2019. In our study, this answer was given only in 33% of cases. Despite many programs aimed at encouraging self-examinations, most of our participants reported never performing them.

In 2013, Góralaska conducted a similar study of 99 patients aged 15–55 to assess risk factors for cutaneous melanocytic moles and melanoma in patients presenting to dermatologists [22]. The aim of this study was the assessment of risk factors for cutaneous melanocytic moles and melanoma in patients presenting to a dermatologist for control and assessment of patients’ knowledge of these factors. In the study, 99 patients (38 men and 61 women) aged 15–55 years were included. To compare the changes in the distribution of skin cancer risk factors over the last 10 years in Poland, Table 4 compares results from the Goralska study with the current study from changes in the frequency of risk factors.

The data show that over the last 10 years the percentage of patients who do not avoid the sun on sunny days has decreased (34.86% compared to 76% of patients in the study by Góralska et al.—a statistically significant reduction, *p* < 0.005) and the percentage of patients who use sun protection before planned sun exposure has increased (93.23% compared to 81% of patients in the study by Góralska et al.). This is the result of growing awareness of the side effects of intense exposure to UV radiation and the increasing popularity of sunscreens. The approach to sun protection has changed over the years. In the early 20th century, a tan was associated with health and vitality. Around 1945, the first sunscreen products appeared that protected against the sun’s rays. Since then, the perception of sunscreens has changed: initially, they were intended to prevent skin burns during intense exposure to the sun. With the spread of information about skin cancer, sunscreens began to be used for preventive purposes. In recent times, the products have become heavily advertised and have become part of a lifestyle [23]. Łyko and colleagues evaluated sun protection among university students in Poland, among which 60.9% were students of medicine and 96.7% of the responders declared using photoprotection [24].

SPF50 and greater blocks from 98% to 99% of UVB rays. Using sunscreens prevents DNA damage during exposure to solar-simulated radiation (SSR) [24]. However, the correct application density of SPF cream is 2 mg/cm^2^; people usually apply one-third of this value. Therefore, using sunscreens with SPF50 and more may help to provide sufficient UV protection [25]. These data are in concordance with the results of our study because patients who used sunscreen with SPF> 40 did not have more moles.

Over the years, habits regarding spending free time have also changed. Comparing the data from our study and Góralska’s study, the percentage of patients with hobbies related to prolonged sun exposure has significantly decreased [22]. This is reported as a statistically significant reduction—*p*< 0.005 (Table 3). Long-term exposure to UV radiation is associated with skin complications, but also an increased risk of serious eye diseases, including cataracts, corneal degeneration, conjunctival degeneration, and retinal degeneration. A significant portion of UV radiation reaching the eyeball can be eliminated by using sunglasses that block UVR radiation up to 400 nm (filter 99–100% of UVR radiation) [26]. The results of our study show that patients are aware of the harmful effects of exposure to UV radiation on the eyes—only less than one-fifth of patients reported that they never wear sunglasses. Recent studies showed that culture and sex may influence this, a cross-sectional study in the US revealed that Caucasians and women were more likely to wear sunglasses [27]. Moreover, a systematic review showed that sunglasses were the most common choice of sun protection among outdoor workers [28]. Our study showed that in recent years the percentage of people with jobs involving long-term exposure to the sun has slightly decreased. Our data indicate that 8.63% of respondents reported working in conditions of high exposure to the sun, while according to Góralska et al., in 2013 this was 10% [22]. This difference, however, is not statistically significant (*p* > 0.005).

Sunburn, resulting from excessive UV exposure, is the most significant clinical risk factor for melanoma, and the more burn the greater the risk [26]. Moreover, there is a link between childhood sunburn and the risk of both MM and NMSC, indicating that screening and prevention of childhood sunburn could aid in the early detection and reduced risk of MM and NMSC [27]. The comparative study among parents of U.S. adolescents between 1998 and 2004 showed trends in skin cancer risk behaviors, including sunburn and sun protection [28]. In 2004, 47% of people reported getting sunburned during the summer, which is lower than the results from our group of patients (62.27%). Lately, a cross-sectional study from the US conducted between 2010 and 2020, found that the prevalence of sun-protective behaviors and sunburn avoidance among US adults has significantly increased [29].

Literature data indicate that indoor tanning increases the risk of early melanoma and non-melanocytic skin cancers. Furthermore, there is a dose–response relationship between first exposure at an early age and frequency of exposure [30]. Due to increasing knowledge about the harmful effects of UV radiation and the popularity of sun protection, the frequency of using solariums has decreased. While in 2013 10% of surveyed patients used solariums, among our respondents this number dropped to 7.28% [22]. Similar trends of decreasing use of solariums and tanning beds are observed all over the world [31].

Rodriguez-Acevedo et al. conducted a systematic review and meta-analysis of the prevalence of indoor tanning from 2009 to 2018 [32]. This review was compared with the results of a previous meta-analysis by Wehner et al. [33]. The prevalence of past-year indoor tanning among adolescents from 2009 to 2018 was 6.5% (95% CI: 3.3–10.6) in the period 2013–2018 [32], compared with 22% (95% CI: 17.2–26.8) observed for the period 2007–2012 by Wehner et al. [33]. This was reported as a statistically significant reduction of 70%. In the years 2007–2012, the percentage of adults using solariums was 18.2% [33] while in the years 2013–2018 it was 10.4%, which is not a statistically significant change.

Previous studies indicate that a family history of melanoma is a strong risk factor for melanoma development [3]. In the present study, 4.23% of patients reported a positive family history of melanoma. This is lower compared to the data from the study by Góralska et al., in which 9% of respondents declared a family history of melanoma [22]. This difference may be due to a larger percentage of people who have no family history of melanoma coming for preventive skin examinations, motivated by information campaigns rather than fear.

Risk factors for skin melanoma have been studied many times and are well known. One of them is the presence of atypical moles on the patient’s skin. Numerous retrospective studies have noted that the risk of melanoma increases with increases in the number of atypical moles [34,35,36]. Individuals with Fitzpatrick skin types I and II have lighter skin tones and are more prone to sunburn. On the other hand, sun exposure, along with genetic susceptibility, influences the risk of presenting with atypical moles [36]. Moreover, the incidence of atypical nevi in patients with Fitzpatrick skin types I and II is higher compared to those with skin types III and IV, which may support the role of UV radiation in increasing the risk of developing atypical nevi [37]. The results of our study indicate a significantly increased risk of developing atypical moles in patients who exhibited behaviors associated with an increased risk of sun exposure, i.e., sunburn during sunbathing, sun-dependent hobbies, no sun exposure limitation, using a tanning bed, and overlap with melanoma risk factors. UV radiation increases the risk of not only pigmented malignancies but also non-pigmented malignancies and solar keratosis. Similar risk factors for atypical moles and actinic keratosis explain the increased risk of their co-occurrence [38].

Age is a known risk factor for the development of melanoma. The median age at diagnosis is 62 for men and 54 for women, respectively [39]. Atypical moles most often occur in people under 30–40 years old. Their onset of development usually occurs during puberty. Although this is not a typical phenomenon, new atypical moles can appear after age 30. Atypical nevi are dynamic lesions that may change over time, but most of them remain stable throughout a person’s life or regress [40]. Our study showed that the frequency of atypical moles differed in two distinct age groups: 18–45 years and 46 years and older. These results are consistent with the current concept of early onset of atypical moles (below 45 years of age) and their persistence in subsequent years. Moreover, our data indicate that age is not an independent risk factor for the occurrence of atypical moles.

Our study also has its limitations such as other risk factors for the development of atypical nevi and melanoma not considered in this paper: patients’ additional chronic diseases, medications, total lifetime sun exposure, lack of knowledge about the occurrence of melanoma in the family, and occupational UV exposure.

In conclusion, the results of our study indicate a growing awareness of the risk factors of atypical nevi and melanoma and a trend toward reducing the negative impact of these factors on the body. Comparing our results to a 2013 paper investigating the prevalence of similar risk factors in the Polish population, we showed an increased prevalence of avoiding excessive sunlight and artificial radiation exposure, an increased prevalence of using SPF creams before increased UV exposure, and a lower prevalence of hobbies involving extended periods of sunlight exposure. Furthermore, we observed a higher percentage of people without a family history of melanoma presenting for follow-up dermatoscopic examinations. Nevertheless, many people still do not attempt to reduce the negative effects of UV radiation on the skin, so further education of patients by doctors is necessary.

## 5. Conclusions

Our study involved a younger group of patients who participated primarily for skin screening purposes. Most participants sought dermatoscopic examinations due to concerns about numerous moles or changes in existing skin lesions, while preventive skin checks were also common. However, many patients did not perform regular skin self-examinations, underscoring the need for better education on the importance of early detection. Risk factors such as family history of melanoma, childhood sunburn, regular use of tanning beds, sun-related hobbies, and prolonged occupational sun exposure were prevalent among the participants. Despite these risks, consistent use of sun protection measures, such as sunscreen and sunglasses, was notably low. Many patients were also unaware of the number of nevi on their skin and had never undergone a dermoscopic examination before participating in the study.

Our study highlights the significant correlation between the occurrence of atypical lesions and various clinical and demographic factors in a relatively young population. Notably, the male gender emerged as a significant risk factor for developing atypical nevi. Additionally, the number of nevi on the skin was directly associated with an increased risk of atypical moles, particularly in individuals with more than 50 moles, where the risk doubled. The presence of actinic keratosis was another crucial factor, showing a threefold increase in the likelihood of co-occurrence with atypical moles. Lifestyle factors such as sunburn history, sun-dependent hobbies, and tanning bed usage were strongly linked to higher risk of atypical lesions. Specifically, frequent use of tanning beds dramatically elevated the risk. Interestingly, while the use of high-SPF sunscreen was not associated with atypical moles in dermatoscopic examination, lower SPF levels were significantly associated with atypical moles. This underscores the importance of adequate sun protection in mitigating the development of atypical lesions.

These findings underscore the necessity for targeted prevention strategies and regular dermatologic screening, particularly for high-risk groups identified in this study. Early intervention and education on sun safety can play pivotal roles in reducing the incidence of atypical moles and potentially preventing malignant transformations.

Approval for the study was obtained from the Ethics Committee (RNN/39/24/KE). The study was conducted under the EU project titled: Melanoma without secrets—examine the moles. Skin cancer prevention program. No. RPDL.10.03.02-10-A009/22.

## Figures and Tables

**Table 1:** Summary of data collected as part of the study based on patient-completed questionnaires and dermatoscopic examinations.

Questions	Responses	Patients (N)
Demographics		
Sex	Female	N = 393 (66.50)
	Male	N = 198 (33.50)
Mean age [mean (SD)]	Female	46.3 (12.077)
	Male	45.2 (12.590)
Reasons for patients to undergo dermatoscopic examination	Many moles; hyperpigmentations on the skin	N = 384 (64.94)
	Preventive skin examination	N = 147 (24.90)
	The mole has changed, or a new skin lesion has appeared	N = 29 (4.91)
	Family history of skin cancer	N = 12 (2.03)
	Fair skin with blue/green eyes	N = 17 (2.88)
	Weakened immune system	N = 2 (0.34)
Perform skin self-examination	No examination	N = 382 (64.74)
	Once every six months	N = 126 (21.36)
	Once a month	N = 43 (7.29)
	Once a week	N = 30 (5.08)
	Everyday	N = 9 (1.53)
Risk factors		
Family history of melanoma	Yes	N = 25 (4.23)
	No	N = 566 (95.77)
Use tanning bed	1–20/year	N = 34 (5.75)
	≥20 per year	N = 5 (0.85)
	No	N = 552 (93.40
Hobbies related to spending time in the sun	Yes	N = 377 (63.79)
	No	N = 214 (36.21)
Use sunscreen during intense exposure to the sun	Always	N = 202 (34.18)
	Sometimes	N = 349 (59.05)
	Never	N = 40 (6.77)
At least 1 year of work involving prolonged periods of sun exposure	Yes	N = 544 (92.05)
	No	N = 47 (8.95)
Dermatoscopy examination before	Yes	N = 120 (20.30)
	No	N = 471 (79.70)
Knowledge about the number of nevi	Yes	N = 214 (36.21)
	No	N = 377 (63.79)
Use sunglasses	Always	N = 199 (33.67)
	Sometimes	N = 291 (49.24)
	Never	N = 101 (17.09)
Sunburn in childhood	No	N = 324 (54.82)
	1–2 times	N = 207 (35.03)
	>2 times	N = 60 (10.15)
Results of dermatoscopy examination	No neoplasm lesions	N = 509 (86.13)
	A lesion suggesting non-melanoma skin neoplasm	N = 20 (3.38)
	A lesion suggestive of melanoma	N = 10 (1.69)
	Suspicious lesion	N = 52 (8.80)

**Table 2:** Associations between questionnaire responses and dermatoscopic examination.

All n (%)			Dysplastic Nevi		OR [95%CI], *p*-Value Adj. OR [95%CI], *p*-Value
			No	Presence	
**Gender**	**Women—ref**	**393 (66.50)**	350 (68.76)	43 (52.44)	-
	Men	198 (33.50)	159 (31.24)	39 (47.56)	1.996 [1.245; 3.201], 0.004 3.146 [1.507; 6.566], 0.002
Number of moles on skin	<25—ref	227 (38.47)	208 (40.86)	19 (23.46)	-
	25–50	229 (38.81)	206 (40.47)	23 (28.40)	1.222 [0.646; 2.312], 0.537 0.752 [0.312; 1.811], 0.525
	51–100	69 (11.69)	57 (11.20)	12 (14.81)	2.305 [1.057; 5.027], 0.036 1.894 [0.611; 5.874], 0.269
	>100	65 (11.02)	38 (7.47)	27 (33.33)	7.778 [3.936; 15.372], 0.000 4.735 [1.602; 13.989], 0.005
Presence of dysplastic nevi	Numerical variable	-	41 (8.06) Patients Mean 0.18 Max 10	61 (74.39) Patients Mean 1.80 Max 20	2.339 [1.823; 3.002], 0.000 1.880 [1.355; 2.608], 0.000
Actinic keratosis	No—ref	569 (96.28)	494 (97.05)	75 (91.46)	-
	Yes	22 (3.72)	15 (2.95)	7 (8.54)	3.074 [1.213; 7.786], 0.018 6.607 [1.315; 33.196], 0.022
Sunburn during sunbathing	No—ref	223 (37.73)	211 (41.45)	12 (14.63)	-
	Yes	368 (62.27)	298 (58.55)	70 (85.37)	6.824 [2.704; 17.220], 0.000 4.130 [2.184; 7.812], 0.000
Sun-dependent hobby	No—ref	382 (64.64)	345 (67.78)	37 (45.12)	-
	Yes	209 (35.36)	164 (32.22)	45 (54.88)	2.559 [1.594; 4.106], 0.000 2.365 [1.166; 4.798], 0.017
Tanning bed	No—ref.	476 (80.54)	430 (84.48)	46 (56.10)	-
	1–20 [years]	72 (12.18)	51 (10.02)	21 (25.61)	3.849 [2.129; 6.959], 0.000 3.666 [1.444; 9.306], 0.006
	>20	43 (7.28)	28 (5.50)	15 (18.29)	5.008 [2.494; 10.054], 0.000 5.522 [1.674; 18.215], 0.005
Sun exposure limitation	Yes—ref.	385 (65.14)	356 (69.94)	29 (35.37)	-
	No	206 (34.86)	153 (30.06)	53 (64.63)	8.186 [3.673; 18.243], 0.000 4.252 [2.603; 6.947], 0.000
Skin sun protection cream	>40 [Factor]—ref.	238 (40.27)	226 (44.40)	12 (14.63)	-
	<10	49 (8.29)	22 (4.32)	27 (32.93)	23.114 [10.295; 51.891], 0.000 26.262 [8.264; 83.456], 0.000
	10–20	106 (17.94)	82 (16.11)	24 (29.27)	5.512 [2.636; 11.525], 0.000 7.429 [2.698; 20.454], 0.000
	21–40	198 (33.50)	179 (35.17)	19 (23.17)	1.999 [0.945; 4.227], 0.069 3.540 [1.268; 9.885], 0.016

**Table 3:** Associations between age and risk of dysplastic nevi.

	Dysplastic Nevi n (%)	No Dysplastic Nevi
Younger	31 (11.79)	232 (88,21)
Older	51 (15.55)	277 (84.45)
Pearson Chi^2^, *p*	0.189	

**Table 4:** Comparison of the frequency of selected risk factors in our study (2024) and in the study by Góralska et al. (2013) [22]. For each feature, the statistical significance of the difference was calculated.

Risk Factor	Data Collected in Our Study (2024) N = 591	Data Collected in Study by Góralska et. al. (2013) [22] N = 99	χ^2^ Test *p*-Value ^1^
Not avoiding sun exposure on sunny days	34.86%	76%	0.0001
Use of tanning bed	7.28%	10%	0.3466
Positive family history of melanoma	4.23%	9%	0.0422
Using sunscreen before sun exposure	93.23%	81%	0.0001
Sun-dependent hobby	35.36%	47.5%	0.0207
Work involving long periods of sun exposure	8.63%	10%	0.1768

^1^ The statistically significant *p*-level was at <0.05.

## Data Availability

Data are contained within the article.

## References

[1] Surdu S. (2014). Non-melanoma skin cancer: Occupational risk from UV light and arsenic exposure. Rev. Environ. Health.

[2] Gordon R. (2013). Skin cancer: An overview of epidemiology and risk factors. Semin. Oncol. Nurs..

[3] Rastrelli M., Tropea S., Rossi C.R., Alaibac M. (2014). Melanoma: Epidemiology, risk factors, pathogenesis, diagnosis and classification. In Vivo.

[4] Long G.V., Swetter S.M., Menzies A.M., Gershenwald J.E., Scolyer R.A. (2023). Cutaneous melanoma. Lancet.

[5] Strona Główna|Krajowy Rejestr Nowotworów. https://onkologia.org.pl/pl.

[6] Carr S., Smith C., Wernberg J. (2020). Epidemiology and Risk Factors of Melanoma. Surg. Clin. N. Am..

[7] De Braud F., Khayat D., Kroon B.B.R., Valdagni R., Bruzzi P., Cascinelli N. (2003). Malignant melanoma. Crit. Rev. Oncol. Hematol..

[8] Carrera C., Marghoob A.A. (2016). Discriminating Nevi from Melanomas: Clues and Pitfalls. Dermatol. Clin..

[9] Pehamberger H., Steiner A., Wolff K. (1987). In vivo epiluminescence microscopy of pigmented skin lesions. I. Pattern analysis of pigmented skin lesions. J. Am. Acad. Dermatol..

[10] Elder D.E. (2010). Dysplastic naevi: An update. Histopathology.

[11] Wensley K.E., Zito P.M. (2024). Atypical Mole [Updated 3 July 2023]. StatPearls.

[12] Farber M.J., Heilman E.R., Friedman R.J. (2012). Dysplastic nevi. Dermatol. Clin..

[13] FotoFinder Systems|Skin Imaging Systems with Cutting-Edge Technology from Bavaria. https://www.fotofinder.de/en/.

[14] Del Marmol V. (2022). Prevention and screening of melanoma in Europe: 20 years of the Euromelanoma campaign. J. Eur. Acad. Dermatol. Venereol..

[15] Dzwierzynski W.W. (2021). Melanoma Risk Factors and Prevention. Clin. Plast. Surg..

[16] Hasle G. (2019). Sunscreen and malignant melanoma. Tidsskr. Nor Laegeforen..

[17] Alonso-Belmonte C., Montero-Vilchez T., Arias-Santiago S., Buendía-Eisman A. (2022). Current State of Skin Cancer Prevention: A Systematic Review. Actas Dermo Sifiliogr..

[18] Brunssen A., Waldmann A., Eisemann N., Katalinic A. (2017). Impact of skin cancer screening and secondary prevention campaigns on skin cancer incidence and mortality: A systematic review. J. Am. Acad. Dermatol..

[19] Abroms L., Jorgensen C.M., Southwell B.G., Geller A.C., Emmons K.M. (2003). Gender differences in young adults’ beliefs about sunscreen use. Health Educ. Behav..

[20] SDG-Raport 2023. https://raportsdg.stat.gov.pl/.

[21] Dubbini N., Puddu A., Salimbeni G., Malloggi S., Gandini D., Massei P., Ferraùto G., Rubino T., Ricci L., Menchini G. (2021). Melanoma Prevention: Comparison of Different Screening Methods for the Selection of a High Risk Population. Int. J. Environ. Res. Public Health.

[22] Góralska A., Błaszczyk J. (2013). Characteristics of risk factors for development of melanocytic naevi and melanoma in patients presented to a dermatologist to assess melanocytic lesions. Dermatol. Rev. Przegląd. Dermatol..

[23] Surber C., Osterwalder U. (2021). Challenges in Sun Protection. Curr. Probl. Dermatol..

[24] Łyko M., Kruzel M., Kuś A., Maj J., Szepietowski J., Jankowska-Konsur A. (2021). Sun protection among university students in Poland: A survey of awareness and attitudes. Adv. Dermatol. Allergol. Postȩpy Dermatol. I Alergol..

[25] Woźna J., Stępka J., Bałoniak A., Adamski Z. (2024). Evaluation of social knowledge on photoprotection and its relationship with education and age in a Polish seaside town during summer holidays. Photodermatol. Photoimmunol. Photomed..

[26] Dennis L.K., Vanbeek M.J., Freeman L.E.B., Smith B.J., Dawson D.V., Coughlin J.A. (2008). Sunburns and risk of cutaneous melanoma, does age matter: A comprehensive meta-analysis. Ann. Epidemiol..

[27] Li Y., Wu J., Cao Z. (2023). Childhood sunburn and risk of melanoma and non-melanoma skin cancer: A Mendelian randomization study. Environ. Sci. Pollut. Res. Int..

[28] Bandi P., Cokkinides V.E., Weinstock M.A., Ward E. (2010). Sunburns, sun protection and indoor tanning behaviors, and attitudes regarding sun protection benefits and tan appeal among parents of U.S. adolescents-1998 compared to 2004. Pediatr. Dermatol..

[29] McKenzie C., Nahm W.J., Kearney C.A., Zampella J.G. (2023). Sun-protective behaviors and sunburn among US adults. Arch. Dermatol. Res..

[30] An S., Kim K., Moon S., Ko K.P., Kim I., Lee J.E., Park S.K. (2021). Indoor tanning and the risk of overall and early-onset melanoma and non-melanoma skin cancer: Systematic review and meta-analysis. Cancers.

[31] Diehl K., Görig T., Greinert R., Breitbart E.W., Schneider S. (2019). Trends in Tanning Bed Use, Motivation, and Risk Awareness in Germany: Findings from Four Waves of the National Cancer Aid Monitoring (NCAM). Int. J. Environ. Res. Public Health.

[32] Rodriguez-Acevedo A.J., Green A.C., Sinclair C., Van Deventer E., Gordon L.G. (2020). Indoor tanning prevalence after the International Agency for Research on Cancer statement on carcinogenicity of artificial tanning devices: Systematic review and meta-analysis. Br. J. Dermatol..

[33] Wehner M.R., Chren M.M., Nameth D., Choudhry A., Gaskins M., Nead K.T., Boscardin W.J., Linos E. (2014). International prevalence of indoor tanning: A systematic review and meta-analysis. JAMA Dermatol..

[34] Garbe C., Krüger S., Orfanos C.E., Büttner P., Weiß J., Soyer H.P., Stocker U., Roser M., Weckbecker J., Panizzon R. (1994). Associated factors in the prevalence of more than 50 common melanocytic nevi, atypical melanocytic nevi, and actinic lentigines: Multicenter case-control study of the Central Malignant Melanoma Registry of the German Dermatological Society. J. Investig. Dermatol..

[35] Halpern A.C., Guerry D., Elder D.E., Trock B., Synnestvedt M. (1993). A cohort study of melanoma in patients with dysplastic nevi. J. Investig. Dermatol..

[36] Bataille V., Grulich A., Sasieni P., Swerdlow A., Newton Bishop J., McCarthy W., Hersey P., Cuzick J. (1998). The association between naevi and melanoma in populations with different levels of sun exposure: A joint case-control study of melanoma in the UK and Australia. Br. J. Cancer.

[37] Friedman R.J., Farber M.J., Warycha M.A., Papathasis N., Miller M.K., Heilman E.R. (2009). The “dysplastic” nevus. Clin. Dermatol..

[38] Malvehy J., Stratigos A.J., Bagot M., Stockfleth E., Ezzedine K., Delarue A. (2024). Actinic keratosis: Current challenges and unanswered questions. J. Eur. Acad. Dermatol. Venereol..

[39] Ingraffea A. (2013). Melanoma. Facial Plast. Surg. Clin. N. Am..

[40] Rezze G.G., Leon A., Duprat J. (2010). Dysplastic nevus (atypical nevus). An. Bras. Dermatol..

